# Supplementary material for: OpenMM 7: Rapid development of high performance algorithms for molecular dynamics
Source: PLoS Comput Biol. 2017 Jul 26;13(7):e1005659. doi: 10.1371/journal.pcbi.1005659 (PMC5549999; doi:10.1371/journal.pcbi.1005659)
Supplement: S1 Source Code — (ZIP) [file pcbi.1005659.s002.zip › OpenMM-7.0.1-Source/docs/api-c++/generated/OpenMM.Continuous3DFunction.html]

Continuous3DFunction — OpenMM @OPENMM\_MAJOR\_VERSION@.@OPENMM\_MINOR\_VERSION@ documentation


# Continuous3DFunction¶

*class* `OpenMM::``Continuous3DFunction`¶
:   This is a `TabulatedFunction` that computes a continuous three dimensional function.

    Methods

    |  |  |
    | --- | --- |
    | `Continuous3DFunction` | Create a `Continuous3DFunction` f(x,y,z) based on a set of tabulated values. |
    | `getFunctionParameters` | Get the parameters for the tabulated function. |
    | `setFunctionParameters` | Set the parameters for the tabulated function. |
    | `Copy` | Create a deep copy of the tabulated function |

    `Continuous3DFunction`(int *xsize*, int *ysize*, int *zsize*, *const* std::vector<double> &*values*, double *xmin*, double *xmax*, double *ymin*, double *ymax*, double *zmin*, double *zmax*)¶
    :   Create a `Continuous3DFunction` f(x,y,z) based on a set of tabulated values.

        |  |  |
        | --- | --- |
        | Parameters: |  |

        - **values** – the tabulated values of the function f(x,y,z) at xsize uniformly spaced values of x between xmin and xmax, ysize values of y between ymin and ymax, and zsize values of z between zmin and zmax. A natural cubic spline is used to interpolate between the tabulated values. The function is assumed to be zero when x, y, or z is outside its specified range. The values should be ordered so that values[i+xsize\*j+xsize\*ysize\*k] = f(x\_i,y\_j,z\_k), where x\_i is the i’th uniformly spaced value of x. This must be of length xsize\*ysize\*zsize.
        - **xsize** – the number of table elements along the x direction
        - **ysize** – the number of table elements along the y direction
        - **ysize** – the number of table elements along the z direction
        - **xmin** – the value of x corresponding to the first element of values
        - **xmax** – the value of x corresponding to the last element of values
        - **ymin** – the value of y corresponding to the first element of values
        - **ymax** – the value of y corresponding to the last element of values
        - **zmin** – the value of z corresponding to the first element of values
        - **zmax** – the value of z corresponding to the last element of values

    void `getFunctionParameters`(int &*xsize*, int &*ysize*, int &*zsize*, std::vector<double> &*values*, double &*xmin*, double &*xmax*, double &*ymin*, double &*ymax*, double &*zmin*, double &*zmax*) *const*¶
    :   Get the parameters for the tabulated function.

        |  |  |
        | --- | --- |
        | Parameters: |  |

        - **values** – [out] the tabulated values of the function f(x,y,z) at xsize uniformly spaced values of x between xmin and xmax, ysize values of y between ymin and ymax, and zsize values of z between zmin and zmax. A natural cubic spline is used to interpolate between the tabulated values. The function is assumed to be zero when x, y, or z is outside its specified range. The values should be ordered so that values[i+xsize\*j+xsize\*ysize\*k] = f(x\_i,y\_j,z\_k), where x\_i is the i’th uniformly spaced value of x. This must be of length xsize\*ysize\*zsize.
        - **xsize** – [out] the number of table elements along the x direction
        - **ysize** – [out] the number of table elements along the y direction
        - **zsize** – [out] the number of table elements along the z direction
        - **xmin** – [out] the value of x corresponding to the first element of values
        - **xmax** – [out] the value of x corresponding to the last element of values
        - **ymin** – [out] the value of y corresponding to the first element of values
        - **ymax** – [out] the value of y corresponding to the last element of values
        - **zmin** – [out] the value of z corresponding to the first element of values
        - **zmax** – [out] the value of z corresponding to the last element of values

    void `setFunctionParameters`(int *xsize*, int *ysize*, int *zsize*, *const* std::vector<double> &*values*, double *xmin*, double *xmax*, double *ymin*, double *ymax*, double *zmin*, double *zmax*)¶
    :   Set the parameters for the tabulated function.

        |  |  |
        | --- | --- |
        | Parameters: |  |

        - **values** – the tabulated values of the function f(x,y,z) at xsize uniformly spaced values of x between xmin and xmax, ysize values of y between ymin and ymax, and zsize values of z between zmin and zmax. A natural cubic spline is used to interpolate between the tabulated values. The function is assumed to be zero when x, y, or z is outside its specified range. The values should be ordered so that values[i+xsize\*j+xsize\*ysize\*k] = f(x\_i,y\_j,z\_k), where x\_i is the i’th uniformly spaced value of x. This must be of length xsize\*ysize\*zsize.
        - **xsize** – the number of table elements along the x direction
        - **ysize** – the number of table elements along the y direction
        - **zsize** – the number of table elements along the z direction
        - **xmin** – the value of x corresponding to the first element of values
        - **xmax** – the value of x corresponding to the last element of values
        - **ymin** – the value of y corresponding to the first element of values
        - **ymax** – the value of y corresponding to the last element of values
        - **zmin** – the value of z corresponding to the first element of values
        - **zmax** – the value of z corresponding to the last element of values

    Continuous3DFunction \*`Copy`() *const*¶
    :   Create a deep copy of the tabulated function

High performance molecular simulation on GPUs


### Navigation

- Core Objects
- Forces
- Integrators
- Extras

©2015, Stanford University and the Authors.
|
Powered by Sphinx 1.3.1
& Alabaster 0.7.7
|
Page source
